# Supplementary material for: Comparison of Body Characteristics, Carotenoid Composition, and Nutritional Quality of Chinese Mitten Crab (Eriocheir sinensis) with Different Hepatopancreas Redness
Source: Foods. 2024 Mar 25;13(7):993. doi: 10.3390/foods13070993 (PMC11011956; doi:10.3390/foods13070993)
Supplement: Supplementary file 1 [file foods-13-00993-s001.zip › foods-2889156-supplementary.pdf]

Comparison of Body Characteristics, Carotenoid Composition, and Nutritional Quality of Chinese Mitten Crab (*Eriocheir sinensis*) with Different Hepatopancreas Redness

Honghui Guo [1], Jingang Zhang [1,2], Yidi Wu [1], Xiangzhong Luo [1], Zhiqiang Xu [3], Jianlin Pan [3], Guiwei Zou [1] and Hongwei Liang [1,2,*]

[1] Yangtze River Fisheries Research Institute, Chinese Academy of Fishery Sciences, Wuhan 430223, China;

[2] College of Fisheries and Life Science, Shanghai Ocean University, Shanghai 201306, China

[3] Key Laboratory of Freshwater Crustacean Genetic Breeding and Cultivation (Co-Construction by Ministry and Province) Ministry of Agriculture and Rural Affairs, Freshwater Fisheries Research Institute of Jiangsu Province, Nanjing 210017, China;

*** Correspondence:**

Dr. Hongwei Liang

Address: Yangtze River Fisheries Research Institute, Chinese Academy of Fishery Sciences, Wuhan, Hubei, 430223, China

E-mail: lianghw@yfi.ac.cn (H.L.)

Office tel: 86-27-81780097

Material and Methods

Text S1. Hepatopancreas and gonads paraffin section analysis

Hepatopancreas and gonads samples were cut as size as 3—5 mm^3 and fixed in 10% neutral-buffered formalin. After 48-hour fixation, they were processed routinely including embedded in paraffin wax, sectioned (5 μm), and stained with haematoxylin and eosin (H&E). Histopathological assessment was done on a light microscopy (Nikon H600L Microscope and image analysis system, Tokyo, Japan).

Text S2. Total sugar, lipid and protein analysis

The detection protocol of total sugar levels in muscle, hepatopancreas and gonads were described as followed. In brief, the samples (muscle, hepatopancreas, and gonads) were weighted, and added into conical bottle with 50 mL water and 15 mL concentrated hydrochloric acids. Then they were hydrolyzed in 100℃ water for 3 h and cooled to room temperature after that. And filtered and filled with water to 250 mL. The absorb of each samples were tested at 490 nm. The total sugar contents were calculated by standard curve. The detection protocol of the lipid content was described as followed. In brief, 4 g sample was added into a 50 mL tube with 8 mL water and 10 mL hydrochloric acid, then the tube was placed in a water (70—80℃) until the sample was fully digested. Subsequently, 10 mL ethanol was added then 25 mL anhydrous ether was added until the upper liquid clear, and carefully absorbed the supernatant after that. Finally, the anhydrous ether was recovered and dried until the constant weight (the difference between the two weights shall not exceed 2 mg). The detection protocol of the protein concentrations were described by the Kjeldahl method , using a 6.25 nitrogen-to protein conversion factor.

Text S3. Carotenoid compositions analysis

In brief, 0.2 g of samples were added into 4 mL acetone, and homogenized for 15 min in ultrasonic cleaner for dissociation of the pigment. Then homogenates were centrifuged (4°C, 2237 xg) for 10 min, and the supernatant removed. Re-extracted using acetone until colorless in dark. Total carotenoids were analyzed by spectrophotometry at 478 nm against an acetone blank, and the concentration of total carotenoids estimated based on the commercially available standards of β-carotene in acetone. A part of the carotenoid extracted from each sample was used to analyse free carotenoids. The samples were filtered through a 0.45 μm syringe

filter before High-perpormance liquid chromatography (HPLC) analysis. Carotenoids were identified based on the five commercially available standards of known concentration (Astaxanthin, lutein, zeaxanthin and β-carotene-Shanghai yuanye Bio-Technology Co., Ltd, Shanghai, China; Canthaxanthine-Dr. Ehrenstorfer GmbH, Augsburg, Germany. Quantitative determination was performed using an external standard method.

Text S4. Amino acid and fatty acid analysis

The detection protocol of amino acids concentrations were described as followed. Approximately 0.5 g of each sample was weighed into a 50 mL centrifuge tube and homogenized at 10000 rpm for 1 min (30 s × 2 times) in 15 mL of 5% TCA. Samples were gently kept for 2 h after ultrasonication for 5 min in cooling bath, and supernatant was collected by centrifugation at 12,000 × g, 4°C for 10 min. The pH of the supernatant was adjusted to 2.0 using 1M and 6 M NaOH. The solution was made up to the volume of 25 mL with ultrapure water, and filtered through a 0.22 um syringe filter for the quantitative analysis using an amino-acid analyzer (L-8800, Hitachi Co., Ltd., Tokyo, Japan). Sample solutions were separated using an Inertsil ODS-3 C18 column (4.6 mm × 150 mm, 7 μm, GL Sciences Inc. Tokyo, Japan).

The detection protocol of fatty acids were described as followed. All samples were repeated three times. Crude lipids extracted from *E. sinensis* edible tissues were further processed for fatty acid analysis. The results are presented as the percentage of each fatty acid with respect to the total fatty acids (%). The preparation of fatty acid methyl esters (FAMEs) from the crab lipids involved the method of Wen et al. (2001), with slight modification. In brief, saponification was performed using potassium hydroxide (1 mol/L) in methanol at 60 °C for 30 min. After cooling to room temperature (25 °C), sulfuric acid (2 mol/L) in methanol was added for further transesterification at 60 °C for 30 min. The upper organic layer was diluted with n-hexane (5 mL) after centrifugation at 10,000 rpm for 5 min at room temperature (25 °C), and a 0.22 μm filtration membrane was used to remove impurities for analysis. FAMEs were determined using a gas chromatograph (Agilent-2890A, Agilent Technologies Co., Ltd., Santa Clara, CA, USA) with a flame ionization detector. Separation was conducted in a capillary column (30 m, 0.25 mm, 0.25 μm) (DB-WAX, Agilent Technologies Co., Ltd.) with split injection (10:1) and helium (> 99.99%) at a constant flow of 0.8 mL/min. The detector temperature was set to 250 °C, and the injector temperature was 230 °C. The temperature profile of the oven was 100 °C for 1 min, increased by 20 °C/min to 200 °C for 1 min, and then increased by 3 °C/min to 230 °C for 12 min. Identification was accomplished by comparing

the retention times with the standards, and the results are expressed as the relative weight percentage of the identified fatty acids based on the peak areas obtained using the software from the instrument.

Results

Table S1. Spearman correlation coefficients (*r*) among the color of cuirass, abdomen, hepatopancreas, and ovaries, as well as the compositions of carotenoids in hepatopancreas and ovaries in female crabs. Analysis was conducted separately with eight samples. "*" shown significantly correlation between parameters at p <0.05.

		Cuirass			Abdomen			Hepatopancreas								Ovary								
		*L**	*a**	*b**	*L**	*a**	*b**	*L**	*a**	*b**	Lutein	Zeaxanthin	Canthaxanthin	β-carotene	Total carotenoids	*L**	*a**	*b**	Astaxanthin	Lutein	Zeaxanthin2	Canthaxanthin	carotene	Total carotenoids
	*L**	1.00																						
Cuirass	*a**	-0.34	1.00																					
	*b**	-0.21	0.29	1.00																				
	*L**	0.44	-0.27	0.00	1.00																			
Abdomen	*a**	-0.18	0.37	-0.12	0.12	1.00																		
	*b**	0.07	0.06	0.14	0.49	0.62*	1.00																	
	*L**	0.30	0.04	0.28	-0.24	-0.15	0.01	1.00																
	*a**	-0.38	0.67*	-0.28	-0.37	0.43	-0.24	-0.14	1.00															
	*b**	0.01	0.63*	0.18	-0.33	0.21	-0.18	0.26	0.64*	1.00														
	Lutein	-0.22	0.19	-0.28	-0.33	0.07	-0.27	-0.57*	0.39	0.09	1.00													
Hepatopancreas	Zeaxanthin	-0.33	0.06	-0.49	-0.41	0.07	-0.34	-0.51*	0.547*	0.23	0.77*	1.00												
	Canthaxanthin	-0.10	0.40	-0.38	-0.48	0.20	-0.23	-0.05	0.68*	0.51*	0.58*	0.70*	1.00											
	β-carotene	-0.26	0.47	-0.39	-0.34	0.46	-0.20	-0.19	0.86*	0.52*	0.52*	0.69*	0.75*	1.00										
	Total carotenoids	-0.24	0.46	-0.39	-0.33	0.43	-0.21	-0.20	0.86*	0.54*	0.51*	0.70*	0.76*	0.99*	1.00									
	*L**	0.01	0.49	-0.10	-0.03	-0.08	-0.12	-0.37	0.28	0.36	0.44	0.34	0.48	0.31	0.33	1.00								
	*a**	-0.09	0.23	-0.20	0.05	0.44	0.04	-0.34	0.39	0.27	0.32	0.40	0.33	0.70*	0.69*	0.37	1.00							
	*b**	0.31	0.51*	0.33	0.03	0.13	0.23	0.16	0.14	0.58*	0.16	0.04	0.29	0.18	0.19	0.45	0.33	1.00						
	Astaxanthin	0.36	-0.11	0.45	-0.14	-0.26	-0.04	0.27	-0.39	0.08	-0.09	-0.28	-0.01	-0.32	-0.32	0.03	-0.18	0.27	1.00					
Ovary	Lutein	0.22	0.39	0.61*	-0.12	-0.02	0.09	0.19	-0.04	0.43	0.07	-0.16	0.20	-0.05	-0.05	0.29	0.05	0.71*	0.81*	1.00				
	Zeaxanthin	0.15	0.33	0.45	0.04	0.26	0.46	0.16	-0.09	0.23	-0.24	-0.34	-0.02	-0.16	-0.15	0.06	0.07	0.62*	0.55*	0.75*	1.00			
	Canthaxanthin	0.55*	-0.24	0.34	0.02	-0.41	-0.08	0.42	-0.54*	-0.12	-0.32	-0.55	-0.24	-0.47	-0.46	-0.11	-0.21	0.18	0.85*	0.59*	0.51*	1.00		
	carotene	0.67*	-0.03	0.39	0.34	-0.06	0.38	0.49	-0.47	0.04	-0.53*	-0.71*	-0.26	-0.48	-0.48	-0.07	-0.21	0.46	.69*	0.64*	0.72**	0.80*	1.00	
	Total carotenoids	0.34	-0.13	0.30	-0.10	-0.15	0.12	0.37	-0.52*	-0.09	-0.32	-0.55*	-0.52*	-0.61*	-0.62*	-0.29	-0.58*	0.00	0.38	0.18	0.24	0.45	0.46	1.00

Table S2. Spearman correlation coefficients (*r*) among the color of cuirass, abdomen, hepatopancreas, and testicles, as well as the compositions of carotenoids in hepatopancreas in male crabs. Analysis was conducted separately with eight samples. "*" shown significantly correlation between parameters at $p < 0.05$.

		Cuirass			Abdomen			Hepatopancreas								Testicle		
		*L**	*a**	*b**	*L**	*a**	*b**	*L**	*a**	*b**	Lutein	Zeaxanthin	Canthaxanthi n	β-caroten e	Total carotenoids	*L**	*a**	*b**
Cuirass	*L**	1.00																
	*a**	0.46	1.00															
	*b**	0.59*	0.59*	1.00														
Abdomen	*L**	0.00	0.34	0.03	1.00													
	*a**	0.06	0.41	0.08	-0.42	1.00												
	*b**	0.14	0.18	0.14	-0.61*	0.83*	1.00											
Hepatopancreas	*L**	0.25	-0.01	0.16	-0.24	0.10	0.01	1.00										
	*a**	-0.07	0.02	-0.10	-0.34	0.50*	0.29	0.04	1.00									
	*b**	-0.04	0.11	0.25	-0.24	0.06	-0.12	0.41	0.54*	1.00								
	Lutein	-0.33	-0.36	-0.04	-0.16	0.02	-0.06	-0.21	0.37	0.41	1.00							
	Zeaxanthin	-0.13	-0.01	-0.08	0.22	-0.09	-0.25	-0.12	0.34	0.38	0.582*	1.00						
	Canthaxanthin	-0.14	0.42	-0.01	0.53*	0.09	-0.17	-0.14	0.37	0.26	0.10	0.52*	1.00					
	β-carotene	-0.12	0.12	0.03	0.47	-0.17	-0.41	-0.26	0.36	0.34	0.57*	0.69**	0.52*	1.00				
	Total carotenoids	-0.18	0.03	-0.05	0.55*	-0.27	-0.45	-0.36	0.29	0.24	0.59*	0.61*	0.55*	0.96**	1.00			
Testicle	*L**	0.01	-0.17	0.22	-0.44	0.17	0.42	0.23	-0.11	-0.16	0.10	-0.29	-0.47	-0.34	-0.30	1.00		
	*a**	-0.21	-0.27	0.07	-0.28	-0.10	0.09	-0.12	0.10	0.00	0.27	0.05	0.12	-0.04	-0.02	0.04	1.00	
	*b**	-0.11	-0.17	0.28	-0.37	-0.11	0.07	0.21	-0.09	0.07	-0.04	-0.37	-0.13	-0.30	-0.30	0.15	0.78**	1.00
